# Concept, Design, and Early Prototyping of a Low-Cost, Minimally Invasive, Fully Implantable Left Ventricular Assist Device

**DOI:** 10.3390/bioengineering9050201

**Published:** 2022-05-06

**Authors:** Florin Alexandru Pleșoianu, Carmen Elena Pleșoianu, Iris Bararu Bojan, Andrei Bojan, Andrei Țăruș, Grigore Tinică

**Affiliations:** 1 Department of Surgical Science, ‘Grigore T. Popa’ University of Medicine and Pharmacy, 700115 Iași, Romania; alexandru.plesoianu@umfiasi.ro (F.A.P.); andrei-a-bojan@d.umfiasi.ro (A.B.); 2 Department of Internal Medicine, Faculty of Medicine, ‘Grigore T. Popa’ University of Medicine and Pharmacy, 700115 Iași, Romania; 3 Department of Clinical Cardiology, ‘Prof. Dr. George I.M. Georgescu’ Institute of Cardiovascular Diseases, 700503 Iași, Romania; 4 Department of Pathophysiology, Morpho-Functional Sciences, Faculty of Medicine, ‘Grigore T. Popa’ University of Medicine and Pharmacy, 700115 Iași, Romania; 5 Department of Cardiovascular Surgery, Faculty of Medicine, ‘Grigore T. Popa’ University of Medicine and Pharmacy, 700115 Iași, Romania; andrei.tarus@umfiasi.ro (A.Ț.); grigore.tinica@umfiasi.ro (G.T.); 6 Department of Cardiovascular Surgery, ‘Prof. Dr. George I.M. Georgescu’ Institute of Cardiovascular Diseases, 700503 Iași, Romania

**Keywords:** left ventricular assist device, mechanical circulatory support, advanced heart failure, minimally invasive, axial-flow pump

## Abstract

Despite evidence associating the use of mechanical circulatory support (MCS) devices with increased survival and quality of life in patients with advanced heart failure (HF), significant complications and high costs limit their clinical use. We aimed to design an innovative MCS device to address three important needs: low cost, minimally invasive implantation techniques, and low risk of infection. We used mathematical modeling to calculate the pump characteristics to deliver variable flows at different pump diameters, turbomachinery design software CFturbo (2020 R2.4 CFturbo GmbH, Dresden, Germany) to create the conceptual design of the pump, computational fluid dynamics analysis with Solidworks Flow Simulation to in silico test pump performance, Solidworks (Dassault Systèmes SolidWorks Corporation, Waltham, MA, USA) to further refine the design, 3D printing with polycarbonate filament for the initial prototype, and a stereolithography printer (Form 2, Formlabs, Somerville, MA, USA) for the second variant materialization. We present the concept, design, and early prototyping of a low-cost, minimally invasive, fully implantable in a subcutaneous pocket MCS device for long-term use and partial support in patients with advanced HF which unloads the left heart into the arterial system containing a rim-driven, hubless axial-flow pump and the wireless transmission of energy. We describe a low-cost, fully implantable, low-invasive, wireless power transmission left ventricular assist device that has the potential to address patients with advanced HF with higher impact, especially in developing countries. In vitro testing will provide input for further optimization of the device before proceeding to a completely functional prototype that can be implanted in animals.

## 1. Introduction

“Advanced”, ”refractory”, or ”end-stage” heart failure (HF) describes a clinical syndrome characterized by persistent or progressive symptoms and ventricular dysfunction despite guideline-directed medical therapy [1,2,3,4,5]. Patients with end-stage HF fall into stage D of the ABCD classification of the American College of Cardiology/American Heart Association, and class III–IV of the New York Heart Association functional classification [6].

The prevalence of advanced HF is a real epidemiological challenge with variable reports in different studies and registries, due to the diversity of the definitions used and the populations studied [6]. The prevalence of advanced HF was estimated at 5% of all HF patients by the 2008 Acute Decompensated Heart Failure National Registry (ADHERE) [7], between 6 to 25% in 2011 [8], and 1 to 10% of the total population with HF in 2018 [5]. In 2020, it was reported that of the total population with HF (8.52 cases per 1000 inhabitants) 29% have mild HF, 19% moderate HF, and 51% severe HF [9]. Despite discrepancies regarding the prevalence of advanced HF, there is a unanimous consensus on the increased prevalence of this pathology in the context of an aging population and the increased survival of patients with HF [10].

The individual and socio-economic burden of the disease is extremely high, with estimates suggesting that the total cost of HF in the United States will increase from USD 30.7 billion in 2012 to USD 69.8 billion in 2030 [11]. In 2019, The American Heart Association estimated the cost of each case of HF to be USD 5380 [12], leading to a global economic burden of the disease reaching USD 346.17 billion [9]. Furthermore, the mortality of patients with advanced HF is extremely high [13], with an estimated 1-year survival ranging from 50 to 71.9% [7,14].

All patients for whom medical therapy alone is insufficient to optimize hemodynamics and improve end-organ function should be evaluated for advanced HF therapies: long-term mechanical circulatory support (MCS) or cardiac transplantation [1,3]. Although it remains the gold standard [1], the shortage of donors makes heart transplantation a limited option. Therefore, the focus is being diverted towards MCS devices as potential short- and long-term solutions [15]. The data published by the International Society for Heart and Lung Transplantation show an increase in the use of MCS for pre-transplant patients with a rapid increase from 2007 to 2013 and a relatively constant subsequent use of about 50% [15]. The close follow-up of advanced HF patients is mandatory for appropriate candidate selection and timing for circulatory support [16]. The Interagency Registry for Mechanically Assisted Circulation (INTERMACS) classification system provides a risk stratification to better define the prognosis and urgency of intervention [5,16,17].

MCS has steadily evolved over the past few decades [18,19,20,21], leading to the increased survival and quality of life of patients in advanced HF, as demonstrated by key clinical trials [19,22,23,24,25,26,27,28]. At present, survival rates following the insertion of a left ventricular assist device (LVAD) have reached 81.5% at 1 year and 71.2% at 2 years [29].

The greatest potential impact of MCS on public health is for patients with oral therapies for HF, long before the development of cardiogenic shock and multiple organ dysfunction, in which support could be initiated electively with long-term benefits and diminished perioperative risk [30]. However, important complications [29,31] as well as high costs [32] still limit the clinical use of these devices. The average cost of LVAD-related hospitalization increased from USD 194,380 in 2005 to USD 234,808 in 2011 [33]. A 2017 paper on LVADs cost-effectiveness in France showed that the use of LVAD in patients with end-stage HF yields greater benefits in terms of survival than medical management at an extra lifetime cost exceeding the EUR 100,000/quality-adjusted life year (QALY). Currently, LVADs are included in the basic healthcare package in several countries, such as the USA, the UK, Norway, the Netherlands, and France [34]. Another 2016 study of the cost-effectiveness of LVADs as destination therapy in ambulatory patients with advanced HF showed that LVADs in non-inotrope-dependent HF patients improved quality of life but substantially increased lifetime costs because of frequent readmissions and costly follow-up care (the mean cost of LVAD implantation was USD 175,420). The mean cost of readmission was lower before LVAD than after (USD 12,377 vs. USD 19,465, respectively; *p* < 0.001), while monthly outpatient costs were similar (USD 3364 vs. USD 2974, respectively; *p* = 0.54) [35].

It is also very important to stress the benefits of cardiac rehabilitation in VAD patients to progressively reduce the physical and functional impairments in order to resume meaningful daily activities [36].

While improving survival and quality of life in their recipients, LVADs’ major limitations refer to thrombus formation, hemolysis, infections, gastrointestinal bleeding, and pump malfunction [28,29].

Different LVADs are being designed to address these issues. Computational flow simulations may enhance the information of clinical image data and may have an application in clinical investigations of hemodynamic changes in LVAD-supported patients. [37,38].

In this context, our multidisciplinary team of bioengineers, cardiologists, and cardiovascular surgeons designed an innovative circulatory assist device to support patients with advanced HF. We used mathematical modeling, turbomachinery preliminary design, Computed Fluid Dynamics (CFD) simulation, Computer-Assisted Design, and stereolithography 3D printing, presenting the results of the concept, design, and early prototyping of a minimally invasive, fully implantable LVAD.

## 2. Materials and Methods

### 2.1. Concept Development

Our main objective was the development of a fully implantable, low-cost LVAD based on a holistic and integrative approach. The patient’s needs for autonomy, durability and low risk of infection, the manufacturer’s needs of low cost and reproductible design, and the needs of the healthcare professional regarding easiness of implantation and follow-up were carefully analyzed and addressed after an extensive literature and patent database review.

### 2.2. Mathematical Modeling

Once the type and the range of diameters of the pump were established, we used mathematical modeling to derive the various rotational speeds of different pump sizes for various flow rates.

We introduced the pump dimensions, the flow rates, and the standardized differential pressure of 100 mmHg in the following equations:

- Using Δ*P* = 1.33 × 10^4^ N/m^2^ and blood density *ρ*, we calculated the specific work “*y*”.

(1) $$y=\frac{\Delta P}{\rho }=\frac{1.33\times 10^{4}}{1.06\times 10^{3}}=12.55\frac{m^{2}}{s^{2}}$$

- For different impeller diameters *D* (11–15 mm) and flow rates *V* (1–10 L/min), the specific diameter *δ* was calculated.

(2) $$\delta =1.054\times D\times \frac{y^{\frac{1}{4}}}{V^{\frac{1}{2}}}$$

- For each calculated specific diameter (*δ*), on the Cordier diagram, we graphically estimated [39] the specific speed (*σ*) at the intersection of *δ* with the optimal efficiency interval for axial pumps. After that, the rotation speed of the pump (*n*) was derived:

(3) $$n=\frac{\sigma \times y^{\frac{3}{4}}}{2.108\times V^{\frac{1}{2}}}$$

For each pump size, we identified the flow rate at which the pump works with maximum efficiency according to the “Efficiency Distribution as a Function of Specific Speed for Turbo Machines Diagram” [40].

### 2.3. Preliminary Pump Design

The design operating point, the pump type, and the dimensions developed in the previous steps were the inputs to create a preliminary design of the pump in turbomachinery interactive design software—CFturbo (2020 R2.4 CFturbo GmbH, Dresden, Germany). The geometries of the pump’s components (impeller, stator, inducer, and diffuser) were automatically generated. We adjusted the number of blades and their angles and the length of each element following the flow trajectories and velocities predicted by the software.

### 2.4. Computational Fluid Dynamics

The preliminary pump design developed in CFturbo was exported to Solidworks Flow Simulation, where in silico tests were performed running CFD analysis.

### 2.5. Computer-Assisted Design

Having the conceptual system, the dimensions, and the geometry of the pump, the entire system (pump, motor, cannula, bearings) was designed and prepared for materialization with computer-aided design software Solidworks (Dassault Systèmes SolidWorks Corporation, Waltham, MA, USA) in a variety of combinations and sizes. Photo-realistic renderings were produced from the 3D model with the PhotoView 360 Solidworks add-in.

### 2.6. Early Prototyping 

The initial prototype was printed using a 2.86 mm polycarbonate (PC) filament with a layer resolution of 100 microns and 50% infill support. The second prototype was developed using a stereolithography (SLA) printer (Form 2, Formlabs, MA, USA) with a layer resolution of 25 microns. The post-processing procedure consisted of a 5 min wash in 99.9% isopropyl alcohol and 60 min in an ultraviolet light curing unit at 60 °C for the material to achieve its optimal mechanical properties. After the curing process, the elements were assembled and checked for appearance and tolerance.

## 3. Results

### 3.1. Concept and Design

The device is composed of two systems: the implantable system with the function of pumping blood from the left heart to the arterial system, and the external system that performs the functions of wireless power supply to the implant and communication with the user and caregivers.

After a literature review of the minimally invasive techniques used in MCS, we decided to adopt the pectoral subcutaneous implantation popularized by CircuLite Synergy [41]. This type of implantation allowed us to integrate the wireless power transmission with benefits of simplified surgery and maximal efficiency in wireless power delivery.

**Figure 1:**
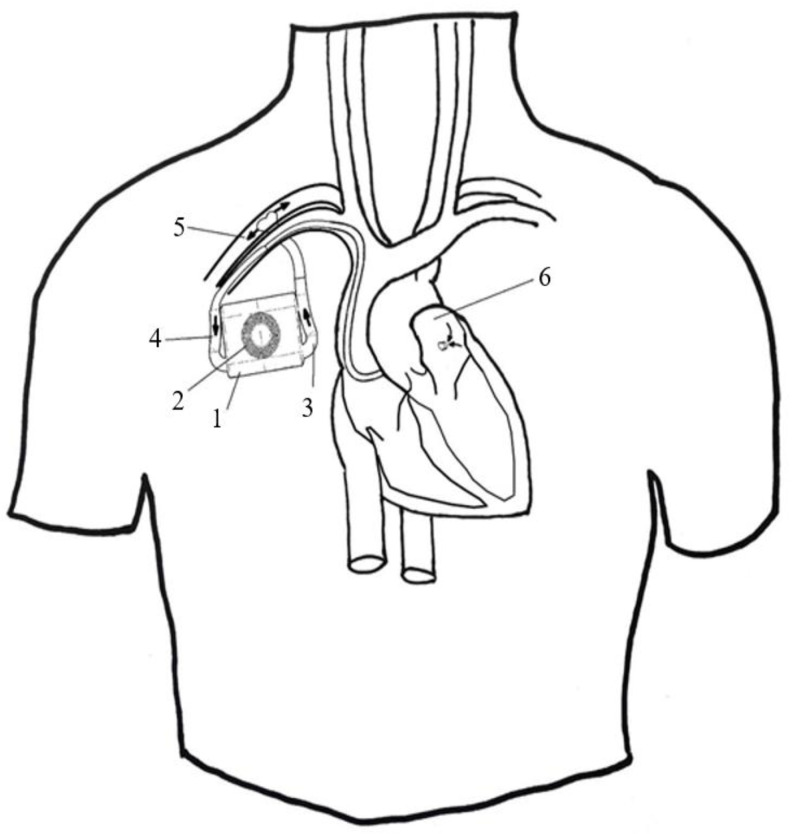
The circulatory assist device—example of implantation in a subcutaneous pocket with inflow cannula connected endovascularly to the left atrium and the outflow cannula in the subclavian artery. Legend: 1: device, 2: antenna, 3: outflow cannula, 4: inflow cannula, 5: subclavian artery, 6: left atrium.

We established the following requirements for the implant: to pump up to 10 L/min of blood at 100 mmHg, to operate autonomously from the external power supply for at least 30 min, to be able to wirelessly receive power from the external power supply, to wirelessly exchange information with external system, to be able to adjust the blood flow according to external controls, and to be biocompatible with tissues in contact. To meet the functional requirements, we decided that the implantable system should contain a housing, a rechargeable battery, an antenna, an electronic circuit, an electrical motor, and a pump. We conceived an innovative, rim-driven, hubless axial-flow pump that can meet the requirements for flow and pressure in the limited space available under the skin in the thoracic (pectoral) area. The outflow cannula of the implant is connected to the left subclavian artery proximal to the implantation incision, while the inflow (aspiration) cannula is connected to the left atrium either via the endovascular approach (Figure 1) or surgery.

To ensure the wireless transmission of power and autonomy, the device was designed with an electromagnetic induction energy antenna and an internal battery.

**Figure 2:**
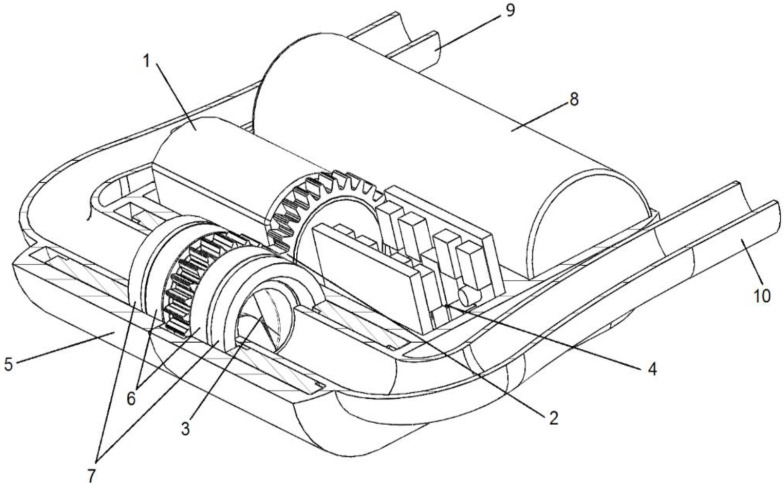
The implantable system of the device—section through the housing. Legend: 1: motor, 2: mechanical transmission, 3: pump rotor, 4: electronic circuit, 5: housing, 6: bearings, 7: sealing elements, 8: battery, 9: outflow cannula, 10: inflow cannula.

The electro-mechanical assembly (Figure 2) is composed of an electric motor and a mechanical gear. The rotational movement of the motor axis is transmitted through the mechanical gear to a tube parallel to the motor. One of the two elements of the transmission, two bearings, and two sealing elements are mounted on the outer surface of the tube. The sealing elements separate the inside of the pump from the rest of the device, preventing contact of the blood with the transmission, the motor, and the electronic circuit. The propeller of the pump is rotated together with the tube with whose internal surface forms a common body, moving the blood from the heart to the subclavian artery. Synthetic grafts connect the device to the heart and the subclavian artery. The surfaces of the pump, the sealing elements, and the housing were designed to be biocompatible (amorphous tetrahedral carbon coating). An external source transmits the energy via electromagnetic resonance to the antenna, and the management unit distributes it for the function of the device as well as for charging the battery.

### 3.2. Mathematical Modeling

**Figure 3:**
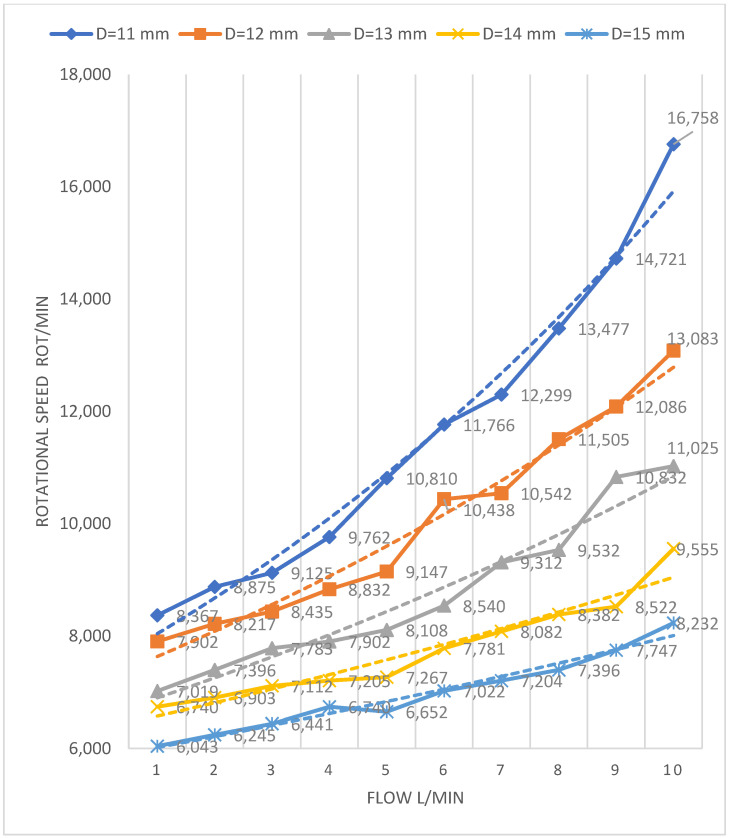
Graphical representation of the values of rotational speeds required for different pump diameters to provide different flow rates calculated by mathematical modeling and approximated on the Cordier diagram. D, diameter.

Considering the results from the previous steps (axial-flow pump, desired flow, and pressure), we used mathematical modeling to calculate the optimal functional/dimensional pump characteristics. As percutaneous implantation constrains the outer diameter of the device, we identified the speed–flow relation for pumps with a diameter of 11 to 15 mm using the equations described in the previous section (Figure 3).

According to the mathematical calculations and graphical approximations on the operation diagrams of the turbomachines [40], the optimal operating parameters for the different diameters of the pumps at a pressure difference of 100 mmHg are shown in Table 1.

### 3.3. The Preliminary Design of the Pump

**Figure 4:**
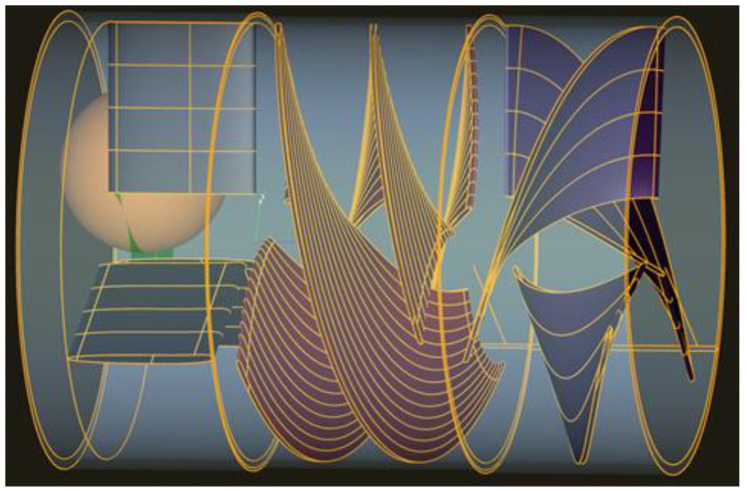
Resulting 3D geometries of the pump. From left to right: inducer (black), impeller (red), diffuser (purple).

CFturbo provided the means to obtain the preliminary design of the pump. In the dialog window of the software, we introduced pump type, operating fluid, total pressure difference, flow rate, and rotational speed. Density and kinematic viscosity were defined as 1060 kg/m^3^ and 3 × 10^−6^ m^2^/s, respectively, and the design operating point was set to Δ*P* = 100 mmHg, *Q* = 10 L/min, and *n* = 16,758 rot/min. The pump inner part consisting of an inducer, impeller, and diffuser was calculated at 13 mm length and 11 mm diameter. The inducer is an inlet stator with three blades parallel with the pump’s axis for directing blood flow (Figure 4). The impeller is a three-blade helical rotor with a wrapped angle of 235° and 225° at the hub and shroud sections of the pump, respectively, that transforms rotational movement into hydrodynamic energy. The diffuser contains three blades that contribute to axial flow of the blood and improve efficiency.

### 3.4. Computed Fluid Dynamics

**Figure 5:**
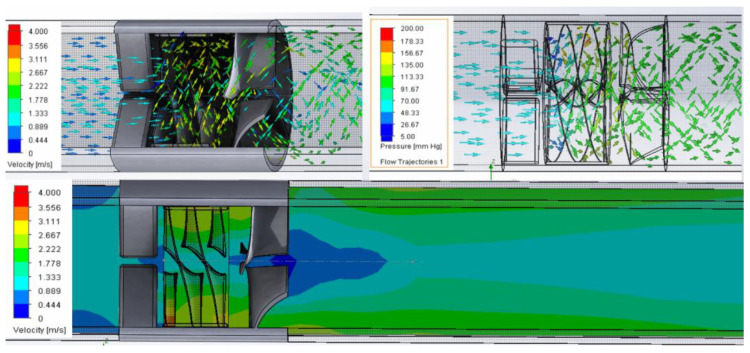
CFD analysis shows flow trajectories (**top**) with velocities (**top left**), pressures (**top right**) and trans-axil plane velocities (**bottom**).

CFD analysis showed that the 11 mm diameter, hubless axial-flow pump was capable of pumping 10 L/min at 100 mmHg with a rotational speed of 16,758 rpm. No evidence of backflow, swirl, or stagnation was observed inside the pump flow trajectories. The rotational pattern of flow was maintained by the diffuser at the outlet. The large gap inside the hubless pump contributed to keeping pressure below 180 mmHg. The internal pump flow trajectories changed from axial to helical patterns, accelerating blood flow up to 3.5 m/s. Low-speed areas were recorded in the mid zone of the inducer and diffuser (Figure 5). The temperature increase was less than 0.1 °C.

### 3.5. Computer-Assisted Design

To pump blood, the impeller must be rotated, which means that the system must contain several components that transform electrical energy into movement, monitor the functionality, communicate with the user, and allow a desired implantation technique.

The pump that will circulate the blood is an innovative hubless, inverted axial-flow pump. Classically, an axial-flow pump has an impeller wrapped around a shaft that is driven by a motor. In contrast, we designed the impeller inside a tube that may be driven by an electrical motor. We took into consideration a solution in which an off-the-shelf electrical motor transmits the torque to the pump by means of a mechanical gear.

**Figure 6:**
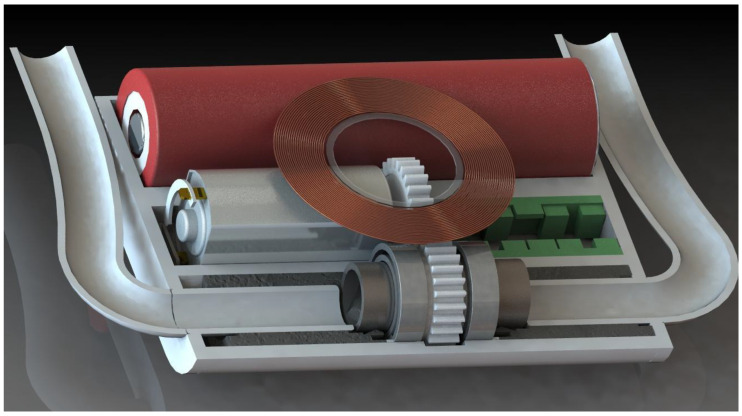
Longitudinal section through the implant including battery, motor, transmission, pump, antenna, suction, and discharge.

The elements were created in CAD software Solidworks (Dassault Systèmes SolidWorks Corporation, Waltham, MA, USA), starting with the impeller and continuing with the other elements around it (Figure 6). All elements were assembled, and a variety of images were rendered from different perspectives and sections. The team analyzed the images and applied the required refinements. Each component was exported as a stereolithographic file. Further optimizations were performed after we analyzed the elements materialized with the 3D printer.

### 3.6. Early Prototyping

**Figure 7:**
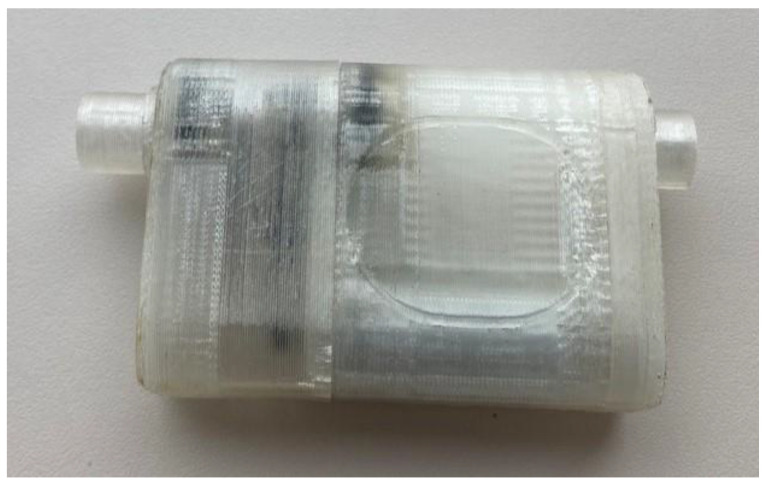
Prototype of the device made using fused deposition modeling technique.

The preliminary design of the pump created in CF-turbo was exported into Solidworks for further refinement and analysis. Different housing geometries were created and exported in .stl format. The first iteration was 3D-printed with a fused filament fabrication technique (Figure 7).

Following the second iteration, the models were materialized at a resolution of 25 microns with clear resin stereolithography technique (Form 2, Formlabs, Somerville, MA, USA).

## 4. Discussion

This device was designed with the aim to meet three main needs in long-term mechanical circulatory supports: low cost, minimally invasive implantation techniques, and low risk of infection.

The device was designed to contain an off-the-shelf motor, bearings, and battery. The electronics, housing, and the pump may be externalized to specialized manufacturers at an estimated low cost. This approach establishes the premises of reducing production costs as well as the need of complex manufacturing facilities that can be a real solution in developing countries where the cost of the current available ventricular assist devices on the market is prohibitive for accessing these therapies [42]. The drawback of the modularity approach is a higher volume compared with a highly integrated variant.

Implantation in a subcutaneous pocket in a single piece similar to an implantable defibrillator without sternotomy or thoracotomy and cardiopulmonary bypass creates the premise for a greater acceptance by both doctors and patients, eliminating the need for a highly invasive intervention, as is the case of on-the-market available devices [43,44]. However, connecting the inflow cannula to the heart endovascularly is a complex intervention and limits the size of the inflow cannula which may allow only partial support of up to 3–5 L/min for an adult patient.

Implantation under the skin creates the premise of wireless energy transmission with high efficiency and low heating of nearby tissues, with the effect of reducing the risk of infection, known as a very important complication in current devices [45,46]. Using transcutaneous energy transfer may present side effects that may occur due to surrounding tissues being heated.

CFD analysis confirmed that pressure and velocity inside the pump should be lower than the hemolysis threshold. The pattern of the helical flow trajectories at the pump outlet suggests that the shape of the diffuser needs further improvement.

There are several limitations related to this project.

There is some concern regarding the interaction between the cardiovascular system and the device in terms of resistance, compliance, and hemodynamics during ventricular interactions. Suction prevention and the avoidance of hemodynamic imbalance between the ventricles need to be addressed and optimized based on pump speed/flow modulation using a software function, which allows hemodynamic monitoring and speed control. Potential for the mechanical failure of the system remains, which we are considering to address based on a compromise between efficiency and safety. The use of modeling and simulation will play an even more critical role at this stage to determine the safety boundary to work within and the adequacy of material thickness to be used. Our in silico simulations confirmed the appropriate performance of the device in terms of flow and pressure generation. Nevertheless, low pressure at the inlet and low flow in mid zone at the outlet has raised awareness of the potential for thrombus formation in these areas. This issue may be addressed with a small hub to reduce the onset of low speed in these areas.

Innovative materials for the device are beyond the scope of this paper.

We propose the design of a ventricular assist device which is still under development. The next stage will focus on in vitro testing to validate its performance in terms of flow visualization and hemolysis level. We expect the test results to provide further input for design optimization. Concerns remain regarding the appropriate flow pattern to achieve bearing wash out and rotor–stator gap to reduce the potential for thrombus formation. We are considering the use of hydro-magnetic, active magnetic, or mechanical bearings to reduce the potential for hemolysis and thrombus formation, although it may lead to a significant cost increase. Although the device is still in its developing phase, we believe it is worth discussing our preliminary results given the innovative approach used. Mathematical formulation, interactive turbo-machinery design, and computational fluid dynamics simulation confirm the feasibility of the proposed concept. The next stage is the acquisition of valuable information to proceed to animal testing.

## 5. Conclusions

We designed a low-cost, minimally invasive, fully implantable mechanical circulatory support device with a rim-driven, hubless axial-flow pump and wireless energy transmission. The device has the potential to address more patients with advanced heart failure, with particular reference to those in developing countries. Further work is needed for device optimization.

## 6. Patents

Patent nr 131433, State Office for Inventions and Trademarks, Romania, 29 March 2019.

## Figures and Tables

**Table 1:** The optimal operating parameters for different pump diameters at a pressure difference of 100 mmHg.

Diameter (mm)	Rotation per Minute (Maximum Efficiency)	Corresponding Flow (Q) (L)
11	15,956	9.9
12	14,615	11.8
13	13,515	13.8
14	12,551	16
15	11,672	18.5

## Data Availability

The data presented in this study are available on request from the corresponding author. The data are not publicly available due to privacy restrictions.
